# Deterioration in hygiene behavior among fifth-year medical students during the placement of intravenous catheters: a prospective cohort comparison of practical skills

**DOI:** 10.1186/s12909-021-02868-5

**Published:** 2021-08-17

**Authors:** Annika Meyer, Jakob Schreiber, Julian Brinkmann, Andreas R. Klatt, Christoph Stosch, Thomas Streichert

**Affiliations:** 1 Department of clinical chemistry, University of Cologne, Faculty of medicine and university hospital, Kerpener Str. 62, 50937 Cologne, Germany; 2 Interprofessional Skills Lab and Simulation center (KISS), University of Cologne, faculty of medicine and university hospital, Joseph-Stelzmann-Straße 9a, 50931 Cologne, Germany

**Keywords:** Hygienic venipuncture, Hygiene, Medical student, Peripheral intravenous catheter (PIV), Objective Structured Clinical Examination (OSCE)

## Abstract

**Background:**

The American Association of Medical Colleges has defined peripheral intravenous cannulation as one of the eight practical skills that a medical student should possess upon graduation. Since following a standard hygiene protocol can reduce the rate of complications such as bloodstream infections, the medical student’s compliance to hygienic standards is highly relevant.

**Methods:**

This unicentric longitudinal cohort study included 177 medical students undergoing OSCE 1 in the winter semesters 2016/2017 and 2017/2018 as well as OSCE 2 during the winter semesters 2018/2019 and 2019/2020 at the University of Cologne. Their performance in peripheral intravenous cannulation was rated by trained student supervisors using a scaled 13-item questionnaire and compared between OSCE 1 and OSCE 2.

**Results:**

Overall, a decline in the correct placement of peripheral intravenous catheters was observed among advanced medical students during OSCE 2 (mean total score: 6.27 ± 1.84) in comparison to their results in OSCE 1 (mean total score: 7.67 ± 1.7). During OSCE 2, the students were more negligent in regard to hygienic behavior, such as disinfection of the puncture site as well as hand disinfection before and after venipuncture. Their patients were also less likely to be informed about the procedure as compared to OSCE 1.

**Conclusions:**

An unsatisfying performance in regard to peripheral intravenous cannulation was observed in medical students with hygiene compliance deteriorating between the third and fifth year of their study. Thus, we promote an extension of practical hygiene and stress management training in medical school to reduce complications associated with intravenous catheters, such as bloodstream infections.

**Supplementary Information:**

The online version contains supplementary material available at 10.1186/s12909-021-02868-5.

## Highlights

- Advanced medical students are less likely to place a PIV correctly.
- Hygiene compliance is especially insufficient in more advanced medical students.
- Patients are less likely informed about PIV by more advanced medical students.

## Background

For over 20 years, the use of intravenous catheters such as peripheral intravenous catheter (PIV), has been determined as one of eight basic practical skills medical students should possess upon graduation by the American Association of Medical Colleges [1, 2]. This is not surprising, since PIV is an established and frequently used method in the clinical practice with 150–330 million applications per year in the USA alone [3, 4].

Despite this, the literature suggests that medical students often gain insufficient practical experience with PIV during their studies. Furthermore, students do not feel confident placing a PIV even though repeated use of a PIV could improve their ability and foster their confidence [1, 5]. Moreover, a study by Friederichs et al. showed the positive effect of simulation training on students’ PIV skills with no special regard to hygiene behavior, but these results did not remain constant over 8 days [6].

There have not yet been any studies specifically evaluating the hygiene behavior of medical students in relation to the use of PIV. Hygiene behavior in regard to PIV is essential to avoid complications, such as PIV-associated bloodstream infections (BSI). With a presumed 250 million cases and an incidence of 21.6 per 1,000 admissions alone in the USA, the rates of nosocomial BSI are rather high [7, 8]. Of all nosocomial BSI, 6.3–23 % are attributed to PIV and their incidence can be reduced by up to 70 % with trained staff and standard hygiene protocols [9–13]. Reducing the rate of nosocomial BSI would not only lessen BSI-associated mortality and morbidity risk, but also reduce health care costs of up to $40,000 per case [9, 14, 15].

 Given their role in the current and future patient care, the relevance of medical students and their compliance with a hygiene protocol when placing a PIV cannot be underestimated.

Consequently, this study seeks to examine hygiene behaviors related to PIV during medical school.

## Material and methods: study design and patient population

This study is an unicentric prospective longitudinal cohort study of 177 medical students undergoing the “objective structured clinical examinations 1 and 2” (OSCE 1 and OSCE 2). Of the original 234 volunteering participants during OSCE 2 in the winter semesters 2018/2019 and 2019/2020, four had to be excluded due to incorrect data collection and 53 students due to missing participation during OSCE 1 in the winter semesters 2017/2018 or 2018/2019.

The OSCEs are intra-curricular practical skill exams, which take place in the third (OSCE 1, formative evaluation) and fifth (OSCE 2, summative test) year of medical school in cologne, to prove practical suitability for clinical internships and the practical year in Germany.

PIV placement is regularly tested in the third year of medical study using OSCE 1 For the purpose of this study, the test station for PIV was additionally set up during OSCE 2 which takes place during the fifth year of medical study. Participation was voluntarily. All members were informed that their participation and the results of the study would not be included in the score of OSCE 2 All data was collected pseudonymized.

Data was collected during OSCE 2 using the same materials (patient couch, stool, simulation dummy, infusion arm, blanket, pillow, infusion, infusion stand, tray, PIV, plaster, stasis loop, disinfectant, gloves, sharp-safe, swabs) and 13-item questionnaire as in OSCE 1. The items were graded by trained professionals on a scale with at least two categorical possibilities. Poor performance was rated with 0, moderate performance with 0.25 or 0.5 and high performance with 0.5 or 1 point. Overall, students could receive 0.5 points in 6 items and 1 point in 7 items, giving students a possible maximum score of 10.

For the purpose of this study, the weighting of the individual items used for grading OSCE 1 was also retained for OSCE 2. The questionnaire contained the items shown in Table 1 and in the Appendix 1.

The PIV placement had to be performed within 5 min under examination conditions on a simulation manikin in both OSCE 1 and OSCE 2, while being observed by either an examiner or data collector. If the 5 min were exceeded, the test was ended prematurely whilst the test scores were still included in the study.

OSCE 2 consists of 14 test stations while OSCE 1 consists of 7. Thus, OSCE 2 takes twice as long as OSCE 1. While the results of this study were not relevant for the OSCE 2 score, the results of the PIV placement during OSCE 1 were included in the OSCE 1 grade and feedback. The examiners at OSCE 1 were more advanced in their medical studies than the data collectors during OSCE 2 and did not know of this study, while the data collectors were fully informed.

In contrast to OSCE 2, the students received a hygiene course and a course for placing intravenous catheters five months to a few weeks prior to the OSCE 1 exam, while the students in their fifth year of study were able to train their PIV-skills in up to four months of clinical clerkships under non-university-controlled conditions (Appendix 2).

### Statistical Analysis

The Data was analyzed using IBM SPSS Statistics 25. For the descriptive statistics, categorical variables were expressed by frequencies and percentages, while the continuous parameters were described by their mean and standard deviation.

The McNemar test and Chi-square test were used to determine statistically significant differences in the grouped item-categories between OSCE 1 and OSCE 2, while the t-test was used to compare averages of continuous characteristics.

In addition, binary logistic regression was used in the subgroup analyses to investigate the possible association between features of hygiene behavior during OSCE 1 and non-sterile venipuncture in OSCE 2.

## Results

**Fig. 1:**
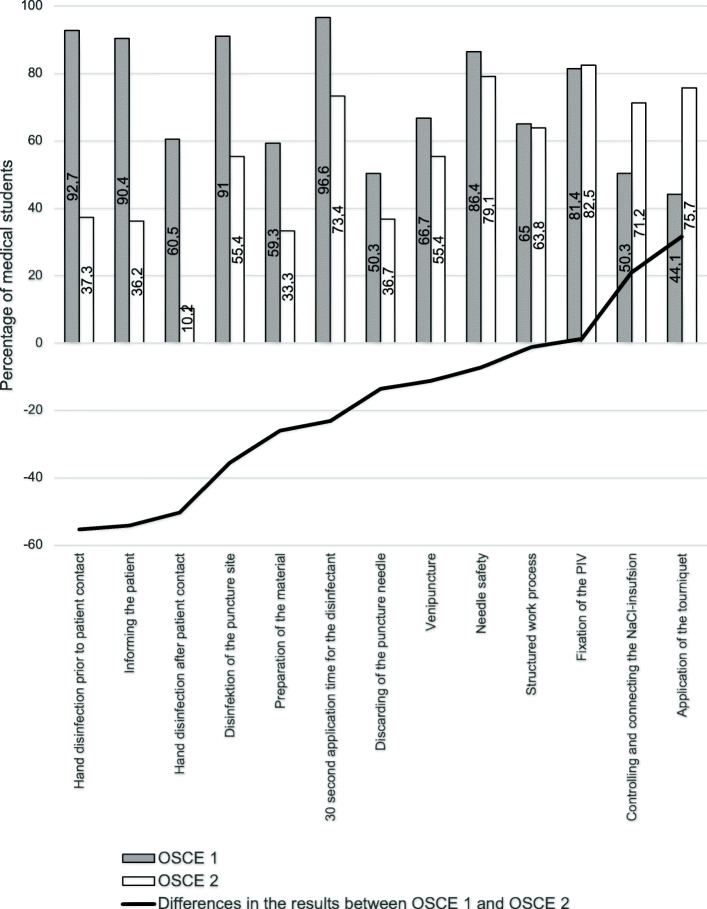
Discrepancy of executing an item during PIV placement correctly between OSCE 1 and OSCE 2. The items were ordered by amount of discrepancy between OSCE 1 and OSCE 2. The grey columns describe the ratio of medical students in their third year (OSCE 1) and the white columns illustrate the medical students in their fifth year (OSCE 2). The difference in their results between OSCE 1 and OSCE 2 is demonstrated by the black line. Frequencies are expressed as percentages

While a total score of 10 for placing a PIV was possible, participants received 6.97 ± 1.91 in average, with fifth-year students scoring worse (6.27 ± 1.84) than in their third year during OSCE 1 (7.67 ± 1.7). Thus, an increase in the number of unsuccessful venipunctures was observed during OSCE 2 (29.4 %) in comparison to OSCE 1 (5.1 %), and the simulation model was less frequently informed about the procedure by the more advanced students (36.2 %) than during OSCE 1 (90.4 %) (Table 1; Fig. 1, Appendix 3–4).

Similar to these results, the fifth-year medical students were also more unsuccessful in hygiene. Hence, hand disinfection prior to patient contact was performed by less than half of the students in OSCE 2 (37.7 %) as compared to the majority of medical students performing it during OSCE 1 (92.7 %). Likewise, the chance of not disinfecting one’s hand after patient contact was higher in OSCE 2 (89.8 %) than during OSCE 1 (39.5 %). Moreover, only 16 medical students (9 %) did not disinfect the puncture site correctly or palpated it after disinfection during OSCE 1, while 79 participants (44.6 %) did not correctly disinfect the puncture site during OSCE 2. In addition, 26.6 % of the medical students in OSCE 2 as compared to 3.4 % of the medical students in OSCE 1 did not obey to the 30-second exposure time of the disinfectant (Table 1; Fig. 1, Appendix 1).

Nonetheless, fifth-year medical students did not score lower on all items. During OSCE 2, the students more often correctly applied the tourniquet (OSCE 1: 44.1 %, OSCE 2: 75.7 %) and were similarly successful in not contaminating nor bending the needle before venipuncture (OSCE 1: 86.4 %, OSCE 2: 79.1 %), in fixation of the PIV (OSCE 1: 81.4 %, OSCE 2: 82.5 %) and structuring their work process (OSCE 1: 65 %, OSCE 2: 63.8 %) in both OSCEs (Table 1).

**Table 1:** Results of the placed PIV in OSCE 1 and 2

N = 177	OSCE 1	OSCE 2	*P*-value
**Score (Maximum = 10)**	7.67 ± 1.707	6.27 ± 1.844	< 0.001**
**Informing the patient**			
The patient is not informed about the procedure	17 (9.6)	113 (63.8)	< 0.001*
The patient is informed about the procedure	160 (90.4)	64 (36.2)	
**Preparation of the material**			
More than one material or the sharp-safe is missing	49 (27.7)	50 (28.2)	< 0.001*
At least one material is missing	23 (13.0)	68 (38.4)	
Complete and correct preparation of the material	105 (59.3)	59 (33.3)	
**Hygienic hand disinfection prior to patient contact**			
No hand disinfection was performed before patient contact	13 (7.3)	111 (62.7)	< 0.001*
Hygienic hand disinfection prior to putting on the medical gloves	164 (92.7)	66 (37.3)	
**Application and deposition of the tourniquet**			
The tourniquet is not applied or disposed of correctly	11 (6.2)	20 (11.3)	< 0.001*
The tourniquet is not applied, while the needle is pulled before disposing of the tourniquet	88 (49.7)	23 (13)	
The tourniquet is applied and disposed in the correct manner	78 (44.1)	134 (75.7)	
**Disinfection of the puncture site**			
The puncture site is not disinfected, or it is palpated after the disinfection and prior to the venipuncture	16 (9)	79 (44.6)	< 0.001*
The puncture site is correctly disinfected	161 (91)	98 (55.4)	
**30 s application time for the disinfectant**			
Disinfectant has not acted for 30 s	6 (3.4)	47 (26.6)	< 0.001*
Disinfectant has acted for 30 s	171 (96.6)	130 (73.4)	
**Venipuncture**			
The vein is not punctured	9 (5.1)	52 (29.4)	< 0.001*
The vein is punctured the second time	50 (28.2)	27 (15.2)	
The vein is punctured the first time	118 (66.7)	98 (55.4)	
**Needle safety while puncturing the vein**			
The needle was touched or bent by the student	24 (13.6)	37 (20.9)	0.079
The needle was neither touched nor bent by the student	153 (86.4)	140 (79.1)	
**Discarding of the puncture needle**			
The needle is not discarded correctly	47 (26.5)	31 (17.5)	< 0.001*
The needle is discarded immediately but not correctly	41 (23.2)	81 (45.8)	
The needle is discarded immediately and correctly	89 (50.3)	65 (36.7)	
**Fixation of the PIV**			
The PIV is not fixated correctly	33 (18.6)	31 (17,5)	0.894
The PIV is fixated correctly	144 (81.4)	146 (82.5)	
**Controlling and connecting the NaCl-infusion**			
The infusion is neither controlled, nor connected to the PIV	44 (24.9)	24 (13.6)	< 0.001*
The infusion is connected to the PIV, without prior control	44 (24.9)	27 (15.2)	
The infusion is controlled and connected correctly to the PIV	89 (50.3)	126 (71.2)	
**Structured work process**			
The work process is not structured	7 (3.9)	22 (12.4)	0.025*
The work process is partly structured	55 (31.1)	42 (23.7)	
The work process is structured	115 (65)	113 (63.8)	
**Hand disinfection after discarding the medical gloves**			
Hands are not disinfected after discarding the medical gloves	70 (39.5)	159 (89.8)	< 0.001*
Hands are disinfected after discarding the medical gloves	107 (60.5)	18 (10.2)	

*Statistically significant difference in this feature between OSCE 1 and 2 was determined using the McNemar-test.

**Statistically significant difference in this feature between OSCE 1 and 2 was determined using the t-test.

## Discussion

With 150–330 million applications per year alone in the USA, the PIV is an established and frequently used method in the clinical practice [3, 4]. Accordingly, the use of PIV is also considered one of the basic practical skills medical students should possess at the end of medical school [1, 2].

Medical students are given the opportunity to place PIV during their internship, as this skill is delegated to them by physicians [1, 16, 17]. Thus, it can be assumed that medical students in higher semesters will have had more opportunities to place PIV during their internships than their less advanced peers, which is consistent to the description of learning-goals in the German national competency-based learning-goal catalogue [18]. Even though more practice evidently leads to more successful PIV placement, the opportunities to place PIV might still be too rare throughout medical school. This hypothesis is also supported by the literature, since 30–60 % of US third-year medical students and 22–23 % of fourth-year medical students have reportedly not the opportunity to place a PIV [1, 5, 19]. This is especially alarming, considering one must place 79 ± 47 PIV to master this skill [20]. However, in a study by Morton et al. only 4 % of students worried about low practice opportunities, while 15–36 % complained about a lack of supervision, feedback, and/or support when placing a PIV [17]. A stronger focus on these aspects in the curriculum would therefore seem necessary. The model curriculum in Cologne covers the leak by inserting a venipuncture-course since 2004 in the first year of medical study followed by a PIV-course in the third year [21]. Thus, the high overall test results in the third as compared to the fifth-year medical students, might be due to the practical training on hygienic placements of PIVs that took place prior to OSCE 1 and not OSCE 2. Similar results in regard to simulation training and successful venipuncture were reported in a study by Friederichs et al., thus demonstrating the relevance of supervised practice opportunities [6].

In accordance with the lower test scores, the patient was less likely informed about the procedure during OSCE 2 compared to OSCE 1. The information provided by the exam coordinator that communication with simple simulation models such as the PIV model would not be assessed during OSCE 2 as opposed to OSCE 1 may have negatively influenced students’ communication skills during OSCE 2 and positively influenced them during OSCE 1. Nevertheless, it seems unlikely this information led to a reduction of up to 64 % in physician-patient communication from OSCE 2 to OSCE 1, especially since the literature suggests a decline in medical student’s communication skills over the course of medical school [22]. Especially the reduction of empathy during medical school seems to negatively influence medical student’s communication skills [23, 24]. Stress and distress are believed to be main factor in the reduction of empathy of medical students [23]. Thus, the stress due to OSCE 2’s relevance, duration and difficulty, as compared to OSCE 1 could have been a reason for the deteriorating doctor patient communication.

A possible solution for better communication skills in medical students during their studies and future career might be voluntary courses for stress management strategies, which have been proven successful for medical students in several studies [25, 26]. Additional curriculum aspects with a positive impact on medical student’s empathy are practical learning, presentation of the patient perspective and early patient contact that had been partially introduced to the Cologne curriculum several years before [27, 28].

Moreover, hygiene during the placement of PIV is highly relevant to minimize the risk of PIV-associated complications, such as PIV-associated BSI. A study by Zhang et al. identified four main ways, contamination of the puncture site (Items: disinfection of the puncture site, hand disinfection), contamination of the PIV (Item: needle safety), contamination of the applied infusion (Item: controlling and connecting the infusion) and hematogenous spread of existing infections, as causal for the development of a PIV-associated BSI [4].

Students performed poorly in the first three causes, while they were particularly negligent in hand disinfection and disinfection of the puncture site during OSCE 1 as compared to OSCE 2. This contradicts a study by Jayarajah et al. in which more advanced medical students were better in hand and equipment hygiene than their less advanced peers. Such differing results may be due to the different study design or country-specific teaching content of the examined medical schools [29].

The average rate of hygienic hand disinfection in students during OSCE 1 (77.5 %) and OSCE 2 (31.3 %) was higher compared to the rate of hygienic hand disinfection in physicians (32 %), nurses (48 %) and medical students (8.5–18.3 %) described in the literature and might be due to the test environment of this study [30, 31].

In a study by Erasmus et al. hand disinfection was perceived as a form of self-protection or self-cleaning and therefore more often performed after (47 %) than before (21 %) contact with the patient [30]. Since model, non-infectious, patients were used during OSCE 1 and OSCE 2, self-protection and self-cleaning might not have been a predominant motivation for the medical students. This might explain the higher rates of hand disinfection before than after patient contact.

Particularly noticeable in this study was the large proportion of students who did not correctly disinfect the puncture site during OSCE 2 as compared to OSCE 1 and thus might be even more likely to contaminate the puncture site with their own skin flora.

Surprisingly, palpation before venipuncture did not increase the success rate of venipuncture in this study. Thus, more insecure medical students might have been more likely to re-palpate the vein before venipuncture, leading to these results.

In general, the number of non-controlled infusions was unsatisfactory with worse scores in OSCE 1. It stands to reason that controlling the infusion before connecting it to the PIV might lower the risk of infection and medication mix-ups. However, further studies have to be conducted to validate this hypothesis.

The set time of five minutes for the PIV placement might not have allowed enough time for hand disinfection after the procedure, disinfection of the puncture site or sufficient ratio of infusion checks. However, in daily clinical practice, five minutes for placing a PIV cannot be guaranteed. According to the literature, an average of 32–120 s is usually sufficient to perform an indwelling venous cannulation [32, 33]. Thus, the argument of insufficient time can be dismissed.

Since the PIV placement was assessed during a graded (OSCE 1) and non-graded (OSCE 2) situation, the students’ ambition and/or the Hawthorne Effect, which also significantly influences the hygienic behavior of physicians (11 %) and nurses (30 %), are alternative explanations for the different hygienic outcomes in OSCE 1 and 2 [34–37]. The Hawthorne effect describes the behavioral adaptation caused by the knowledge of study participants, that they are being observed, which might have been higher during OSCE 1 due to their results being graded [38].

General causes for low compliance in hygiene have been investigated in the literature mainly for hand disinfection but not for disinfection of the puncture site or control of the infusion. However, the causes described in the literature might also act as plausible explanations for the inadequate disinfection of the puncture site and lack of infusion checks in this context.

According to several studies, reasons for low compliance in hand hygiene include lack of knowledge, misinformation, insufficient role models and fear of skin damage caused by disinfection [30, 39–42]. Thus, the older students might have become accustomed to working unhygienically during their often-unobserved placement of PIV in the many months of clinical clerkships or have learned to do so from their role-models on the wards.

However, the most plausible explanation for the better test results in terms of hygiene during OSCE 1 than OSCE 2 is the practical training course in hygienic placement of a PIV held shortly before OSCE 1, which underlines the argument for repetitive hygiene courses for medical students [6, 31, 43, 44].

Another measure to foster hygiene and reduce the contamination load during palpation before venipuncture could be the disinfection of disposable gloves, which showed an improvement in disinfection efficacy in the literature [45].

Nevertheless, further studies have to be conducted to determine the benefit of such disinfection, repetitive courses in hygienic PIV placement and stress management as well as the direct influence of role-modeling on the ward.

## Limitations

In contrary to OSCE 1, participation was voluntary during OSCE 2. Assuming that only students who felt confident in the use of PIV or had a relaxed approach to testing participated in OSCE 2, the data could be biased. Nevertheless, this argument can be refuted by the worse total score in OSCE 2 as compared to OSCE 1.

Even though similar conditions to OSCE 1 were created for the study station in OSCE 2, the time difference between OSCE 1 and OSCE 2 might have caused minimal deviation in examination performance and structure.

The investigators during OSCE 1 and OSCE 2 also differed in both their progress in medical school and knowledge of this study. Since only the investigators of OSCE 2 had knowledge of this study, a detection bias could have been possible during OSCE 2. As according to this study, the focus shifts away from hygiene as medical school progresses, so the investigators of OSCE 1 may have paid less attention to hygiene than the less advanced investigators in OSCE 2. However, if such an effect occurred, it only supports the hypothesis of this study.

Additionally, OSCE 2 may have resulted in a performance bias due to the relevance of its grade, longer duration, and more complex content.

As medical students in higher semesters have more patient contact, they might not place as much value on simulation models. Accordingly, further studies on medical students’ hygiene behaviors directly related to patients need to be conducted.

## Conclusions

Since this study showed, that the communication skills and hygiene behavior of the students in their fifth year of medical students deteriorated significantly compared to their results two years earlier, stress-management and repeating practical hygiene training courses should be additionally implemented in the curriculum to avoid complications such as BSI when placing a PIV.

## Supplementary Information

**Additional file 1:**

**Additional file 2:**

**Additional file 3:**

**Additional file 4:**

## Data Availability

The datasets generated and analyzed during the current study are not publicly available due to sensitivity of human data but are available from corresponding author on reasonable request.

## Abbreviations

PIV: Peripheral Intravenous Catheter
OSCE: Objective Structural Clinical Examination
BSI: Bloodstream Infections
