# Supplementary material for: Deterioration in hygiene behavior among fifth-year medical students during the placement of intravenous catheters: a prospective cohort comparison of practical skills
Source: BMC Med Educ. 2021 Aug 17;21:434. doi: 10.1186/s12909-021-02868-5 (PMC8369648; doi:10.1186/s12909-021-02868-5)
Supplement: Supplementary file 2 — Additional file 2: [file 12909_2021_2868_MOESM2_ESM.pdf]

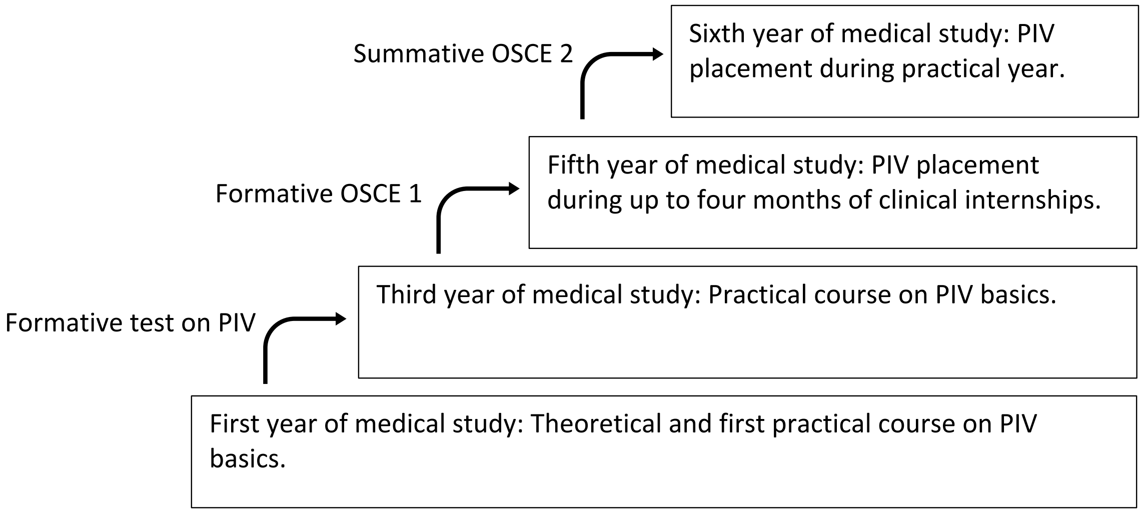

Appendix 2: Chronological overview of the courses and examinations on PIV taken by study participants.
