# Supplementary material for: Deterioration in hygiene behavior among fifth-year medical students during the placement of intravenous catheters: a prospective cohort comparison of practical skills
Source: BMC Med Educ. 2021 Aug 17;21:434. doi: 10.1186/s12909-021-02868-5 (PMC8369648; doi:10.1186/s12909-021-02868-5)
Supplement: Supplementary file 3 — Additional file 3: [file 12909_2021_2868_MOESM3_ESM.pdf]

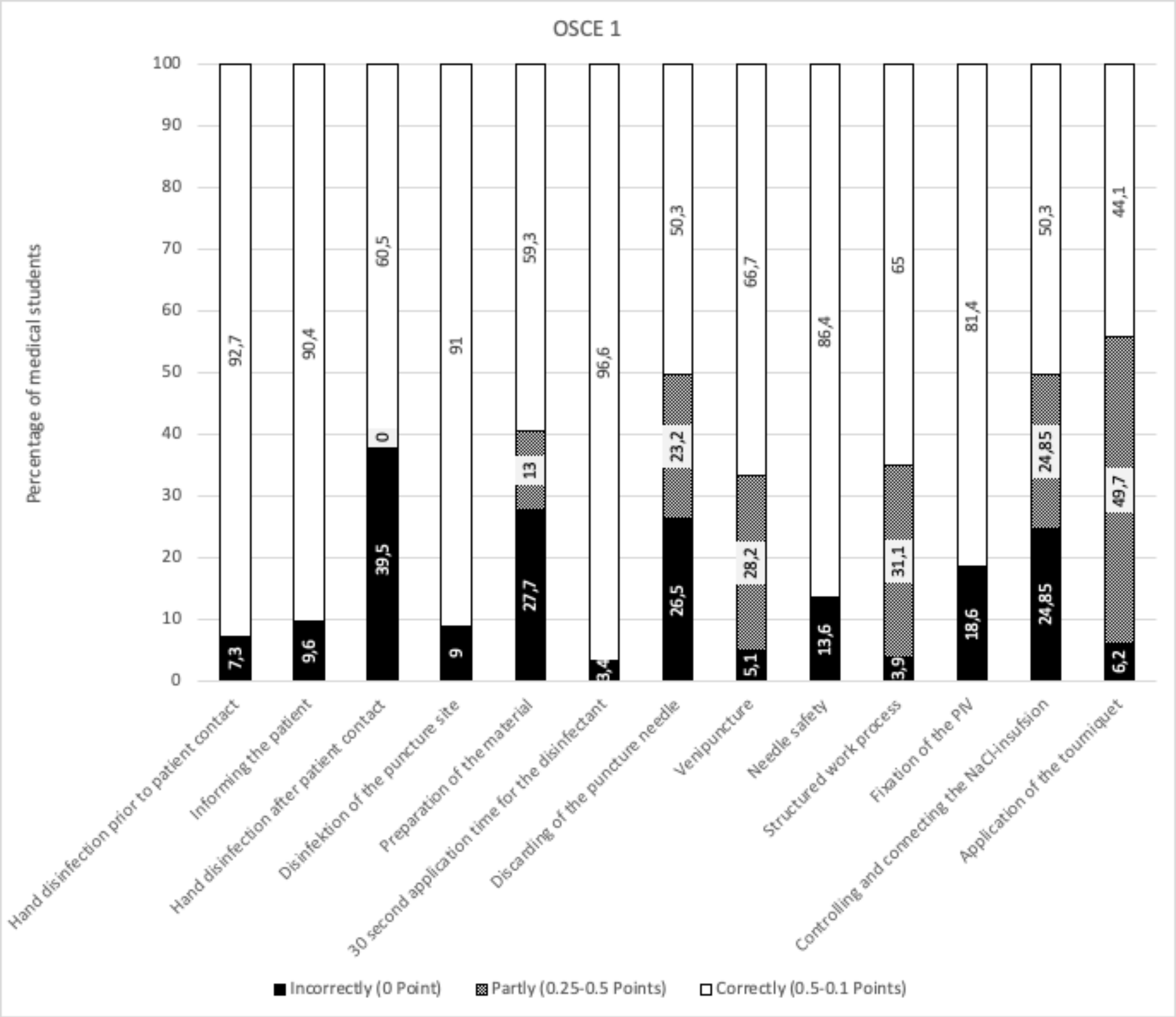

Appendix 3: Results of third-year medical students' placement of PIV during OSCE 1. The items were ordered by amount of discrepancy between OSCE 1 and OSCE 2. White columns demonstrate the percentage of medical students performing the task correctly, while black columns show the ratio of students executing the task incorrectly. The frequency of medical students performing a task partially correct is represented by the grey columns.
